# Loss of PRMT2 in myeloid cells in normoglycemic mice phenocopies impaired regression of atherosclerosis in diabetic mice

**DOI:** 10.1038/s41598-022-15349-6

**Published:** 2022-07-14

**Authors:** Beyza Vurusaner, Prashanth Thevkar-Nages, Ravneet Kaur, Chiara Giannarelli, Michael J. Garabedian, Edward A. Fisher

**Affiliations:** 1 Division of Cardiology, Department of Medicine, Cardiovascular Research Center, New York University Grossman School of Medicine, 435 E. 30th Street, Room 705, New York, NY 10016 USA; 2 Department of Microbiology, New York University Grossman School of Medicine, 450 E. 29th Street, Room 321, New York, NY 10016 USA; 3 Marc and Ruti Bell Vascular Biology Program, New York University Grossman School of Medicine, New York, NY 10016 USA

**Keywords:** Gene regulation in immune cells, Cardiovascular diseases, Diabetes complications

## Abstract

The regression, or resolution, of inflammation in atherosclerotic plaques is impaired in diabetes. However, the factors mediating this effect remain incomplete. We identified protein arginine methyltransferase 2 (PRMT2) as a protein whose expression in macrophages is reduced in hyperglycemia and diabetes. PRMT2 catalyzes arginine methylation to target proteins to modulate gene expression. Because PRMT2 expression is reduced in cells in hyperglycemia, we wanted to determine whether PRMT2 plays a causal role in the impairment of atherosclerosis regression in diabetes. We, therefore, examined the consequence of deleting PRMT2 in myeloid cells during the regression of atherosclerosis in normal and diabetic mice. Remarkably, we found significant impairment of atherosclerosis regression under normoglycemic conditions in mice lacking PRMT2 (*Prmt2*^−/−^) in myeloid cells that mimic the decrease in regression of atherosclerosis in WT mice under diabetic conditions. This was associated with increased plaque macrophage retention, as well as increased apoptosis and necrosis. PRMT2-deficient plaque CD68+ cells under normoglycemic conditions showed increased expression of genes involved in cytokine signaling and inflammation compared to WT cells. Consistently, *Prmt2*^−/−^ bone marrow-derived macrophages (BMDMs) showed an increased response of proinflammatory genes to LPS and a decreased response of inflammation resolving genes to IL-4. This increased response to LPS in *Prmt2*^−/−^ BMDMs occurs via enhanced NF-kappa B activity. Thus, the loss of PRMT2 is causally linked to impaired atherosclerosis regression via a heightened inflammatory response in macrophages. That *PRMT2* expression was lower in myeloid cells in plaques from human subjects with diabetes supports the relevance of our findings to human atherosclerosis.

## Introduction

People with diabetes have more coronary heart disease (CHD) than people without diabetes, even though statin drugs are equally effective in both groups at lowering the blood levels of LDL cholesterol (LDL-C)^1^. Part of the etiology of CHD stems from cholesterol-engorged myeloid-derived macrophages, termed foam cells, that accumulate in arteries, resulting in plaque growth and inflammation. These cells have a diminished capacity to efflux cholesterol, migrate and resolve this inflammation^2^, which are issues that appear to be more pronounced in diabetes^3^. This can ultimately lead to plaques becoming unstable and rupturing, resulting in heart attacks and stroke. Therefore, an important clinical goal to reduce CHD risk is to promote the regression, or resolution, of inflammation in atherosclerotic plaques by returning the foam cells to a healthier state by restoring the aforementioned capacities^4,5^.

In diabetes, this has been particularly challenging, as the molecular mechanisms underlying the attenuation of CHD reduction by LDL-C lowering are incompletely determined. To narrow this gap in understanding, we have identified protein arginine methyltransferase 2 (PRMT2) as a protein whose expression in macrophages was extremely low under high glucose compared to normal glucose conditions^6^. PRMT2 is an enzyme that catalyzes the transfer of a methyl group from S-adenosylmethionine (SAM) asymmetrically to the arginine residues on histones, such as histone H3R8^7,8^, and other proteins to affect their functions^9–11^. In fibroblasts, PRMT2 has been reported to inhibit NF-kappa B-dependent transcription by increasing the nuclear accumulation of I kappa B-alpha^12^ which is a repressor of NF-kappa B. It has also been reported that a reduction in *Prmt2* copy number in lung macrophages increases their LPS responsiveness and promotes inflammatory cytokine expression^13^. Thus, among other effects, reducing PRMT2 expression has the potential to promote inflammation.

Our group has also reported that PRMT2 deficiency was associated with high-glucose inhibition of the LXR-dependent expression of LXR target genes involved in the anti-atherosclerotic macrophage cholesterol efflux and reverse cholesterol transport, including ATP-binding cassette transporter A1 (*Abca1*) and apolipoprotein E (*Apoe*)^6^. LXRs are nuclear receptors that control the expression of genes involved in cholesterol homeostasis, and inflammation and are activated by oxidized cholesterol ligands^14^. We also found that in mice the expression of LXRα was induced in plaque macrophages undergoing atherosclerosis regression^15^ and is required for atherosclerosis regression after reversal of hypercholesterolemia^16^.

As implied above, it should first be noted that in the current study, regression is defined as a reduction in the number or activation state of the inflammatory macrophages that accumulate in atherosclerotic plaques during their progression. Because we have been able to show that diabetes impaired atherosclerosis regression in a mouse model^17^ in a manner consistent with the clinical data cited, we decided to adapt this model to the study of PRMT2. Given that PRMT2 has the potential to repress inflammation and increase cholesterol efflux pathways, we hypothesized that the reduced expression of PRMT2 by high glucose is a key determinant of impaired atherosclerosis regression in diabetes. To test this hypothesis, we examined whether the myeloid-specific deletion of *Prmt2* would impair the regression of atherosclerosis in normoglycemic conditions, thus mimicking the decrease in the regression of atherosclerosis in WT mice under diabetic conditions. In fact, we found that the deletion of PRMT2 in myeloid cells under normoglycemic conditions phenocopied the impaired atherosclerosis regression in hyperglycemic mice by reducing the ability of macrophages to migrate out of the plaque. This was associated with increases in macrophage (CD68+ cells) gene expression linked to cytokine signaling and inflammation. Studies in macrophages in vitro implicate effects on NF-kappa B-mediated inflammation. Thus, the expression of PRMT2 is a key factor in promoting atherosclerosis regression, and conversely, its reduction contributes to impaired atherosclerosis regression. Approaches to increase PRMT2 expression in diabetes/hyperglycemia could facilitate atherosclerosis regression and reduce CHD.

## Methods

### Mouse studies

All experimental procedures were performed in accordance with the US Department of Agriculture Animal Welfare Act and the US Public Health Service Policy on Humane Care and Use of Laboratory Animals and were approved by the New York University School of Medicine’s Institutional Animal Care and Use Committee. The studies with mice reported conform to the ARRIVE guidelines. To power our studies, the sample size was predefined as n = 8 to 10 mice/group. Bone marrow from *WT or Prmt2*^−/−^ mice^12^ was transplanted into 8-week old *Ldlr*^−/−^ mice. After 4 weeks of recovery, the mice were placed on a Western diet (21% [wt/wt] fat, 0.3% cholesterol [Research Diets]) for 20 weeks to allow the development of atherosclerotic plaques. Mice were injected IP with streptozotocin (STZ; 50 mg/kg, Sigma-Aldrich) or citrate buffer for 5 days to induce diabetes mellitus or to serve as a control. Regression was induced by reversal of hyperlipidemia using an apoB-antisense oligonucleotide (ASO) (Ionis Pharmaceuticals, 50 mg/kg; twice a week for 3 weeks) on a chow diet (13% fat kcal, 0% cholesterol). Baseline groups were sacrificed at this time. Mice were randomly assigned to either baseline or 1 of 4 regression groups: apoB-ASO + citrate (nondiabetic) or apoB-ASO + STZ (diabetic) harboring bone marrow from either WT or *Prmt2*^−/−^ donors.

Regression groups were sacrificed after 3 weeks on a chow diet. Mice were anesthetized with xylazine/ketamine, and blood was collected via cardiac puncture for plasma analyses. Mice were perfused with 10% sucrose/saline. Aortic roots were dissected and embedded in optimal cutting temperature compound medium (OCT), frozen immediately and stored at − 80 °C until further use.

### Labeling and tracking of blood monocytes

Circulating blood monocytes were labeled in vivo by retroorbital intravenous injection with 200 µL of 1 µm Flouresbrite YG microspheres (Polysciences Inc, PA) diluted 1:4 in sterile PBS in mice undergoing regression 3 days prior to harvest. The efficiency of bead labeling was verified 24 h later by flow cytometry. The number of labeled macrophages (Ly6Clo) remaining in the aortic root lesions was determined as previously described^4^. Ly6Chi monocytes were labeled by intraperitoneal injection with 250 µL of EdU (4 mg/mL) (5-ethynyl-2′-deoxyuridine) from Life technologies (NY), and mice were euthanized after 5 days to assess baseline recruitment or 21 days to assess for retention. The efficiency of EdU labeling was assessed using Click-IT EdU Pacific Blue Flow Cytometry Assay Kit (Life Technologies, NY) after 24 h, and EdU labeled cells were stained using a Click-IT reaction with Alexa Fluor 647 nm-azide (Click-iT EdU Imaging Kit, Invitrogen, CA).

### Tissue collection and immunohistochemistry

The aortic roots of mice were harvested at baseline, or 3 weeks after apoB-ASO treatment. Hearts were sectioned through the aortic root (6 µm) and stained with hematoxylin and eosin for quantification of lesion area. For immunostaining, slides were fixed with 4% formaldehyde, permeabilized with 0.1% triton, and blocked with 5% BSA. The sections were incubated with antibodies against CD68 (cat # MCA1957, Bio-Rad, CA) to detect macrophages, Ki67 (cat # Ab1666, Abcam, MA) to detect proliferating cells, and cleaved caspase 3 (cat # 9664, Cell Signaling, MA) to detect apoptosis. Sections were then incubated with appropriate secondary antibodies and stained with DAPI to detect nuclei. To distinguish bona fide target staining from background secondary antibody only was used as a control. For collagen quantification, slides were stained with picrosirius red as previously reported^18^, and collagen was visualized using polarizing light microscopy^19^. The images were taken with a Nikon Eclipse microscope and analyzed using Image J software. Quantification of immunostaining was performed from 6 high power fields per aortic root or arch section/mouse. Representative images were selected to represent the mean value of each condition.

### Cell culture

BMDMs were isolated from the tibia and femur of 8–12-week-old male wild type C57BL6J and *Prmt2*^−/−^ mice. Isolated bone marrow cells were treated with red blood cell lysis buffer (Sigma) and re-suspended in differentiation medium (DMEM and l-glutamine with 1 g/L d-glucose + 3.5 g/L l-glucose (normal glucose; NG), or 4.5 g/L d-glucose (high glucose; HG), supplemented with 20% FBS and 10 ng/μL macrophage colony-stimulating factor (M-CSF) (PeproTech, Inc., Rocky Hill, NJ). Cells were passed through a 70 μm filter to clear debris. Following this, cells were plated in 10 cm non-tissue coated plates and allowed to differentiate in either NG or HG containing media for 7 days to obtain non-activated (M0) macrophages. At day 7, the cells were washed in PBS, and re-plated at the desired cell density in a 6-well dish in normal and high glucose media and allowed to attach to the plate. Cells were treated with IL-4 (20 ng/mL) or LPS (100 ng/mL) for the indicated times, and RNA was isolated. For some experiments, NF-kappa B inhibitor, caffeic acid phenethyl ester [CAPE (5 μM)] or TPCA1 (5 μM) was pretreated for 2 h before LPS treatment.

### Plasma cholesterol and blood glucose analyses

Total cholesterol was measured using colorimetric assays (Wako Diagnostics, Richmond, VA). For glucose measurements, mice fasted for 4 h, and glucose in tail blood was measured using a blood glucose monitor (TrueTrack Smart System, Nipro Diagnostics, Inc., Fort Lauderdale, FL).

### Laser capture microdissection and RNA sequencing

CD68+ cells were selected from atherosclerotic plaques by laser capture microdissection (LCM) as previously described^20^. All LCM procedures were performed under RNase-free conditions. Aortic root sections were stained with hematoxylin–eosin. Foam cells were identified under a microscope and verified by positive CD68 staining. For each animal, CD68+ cells were captured from 50 to 60 frozen sections. RNA was isolated using the PicoPure Kit (Molecular Devices, Inc., Sunnyvale, CA), and quality and quantity were determined using an Agilent 2100 Bioanalyzer (Agilent Technologies, Santa Clara, CA). RNA-seq libraries were prepared using the Clontech SMARTer Stranded Total RNA Seq Kit -Pico Input Mammalian following the manufacturer’s protocol. Libraries were purified using AMPure beads, pooled equimolarly, and run on a HiSeq 4000 paired-end reads. FASTQ files were obtained, and low-quality bases, as well as adapter sequences, were trimmed using Cutadapt 1.18. Reads were subsequently mapped to the Mus musculus GRCm38 transcriptome using Kallisto 0.46.2. Raw counts per transcript were summed up at the gene level, and differential expression analysis was performed using edgeR 3.28.0. Genes with a p-value < 0.01 and logFC > 2.0 were determined to be differentially expressed. Pathways analysis was performed using Metascape^21^. RNA sequencing and differential gene expression analysis was performed by the NYU Genome Technology Center.

### RNA isolation and qRT-PCR

Total RNA was extracted using TRIzol reagent (Life Technologies) and Direct-zol RNA MiniPrep columns (Zymo Research) and was reverse transcribed into cDNA using the Verso cDNA Synthesis Kit (Thermo Fisher scientific) according to the manufacturer’s instructions. Quantitative real-time PCR was performed using FAST SYBR Green Master Mix (4385612; Applied Biosystems) on the ABI PRISM 7300 sequence detection system (Applied Biosystems). Cyclophilin A1 was used as a normalization control.

The sequences of the mouse primers used for qPCR are:

*mouse Cyclophilin A1*

F: 5′-GGCCGATGACGAGCCC-3′

R: 5′-TGTCTTTGGAACTTTGTCTGCAA-3′

*mouse Arg 1*

F: 5′-CTGACATCAACACTCCCTGACAAC-3′

R: 5′-CAGATATGCAGGGAGTCACCCAG-3′

*mouse Fizz*

F: 5′-GGTCCCAGTGCATATGGATGAGACC-3′

R: 5′-CACCTCTTCACTCGAGGGACAGTTG-3′

*mouse IL6*

F: 5′-CTGGAAGAGACTTCCATCGAG-3′

R: 5′-AGTGGTATAGACAGGTCTGTTGG-3′

*mouse iNos*

F: 5′-CAGCTGGGCTGTACAAACCTT-3′

R: 5′-CATTGGAAGTGAAGCGTTTCG-3′

### Mouse aortic digestion

*Ldlr*^*−/−*^ mice were placed on a Western diet (42% kcal fat, 0.3% kcal cholesterol, Research Diets) for 18 weeks to develop atherosclerotic plaques. At week 15, mice were injected with STZ (50 mg/kg) or citrate buffer for 5 consecutive days to make the mice either diabetic or nondiabetic. At week 18, post-perfusion aortic arches were harvested and digested in Liberase (5 mg/mL; Roche, 273582), hyaluronidase (99 μg/mL; Sigma, 3506) and DNase I (58 μg/mL; Sigma, DN25) 1 mol/L CaCl_2_ at 37 °C for 15 min at 37 °C using the GentleMacs dissociator (Milteny). The homogenized tissue was filtered using 70 µM filter, washed once with ice cold PBS and centrifuged at 350×*g* for 5 min at 4 °C. The cell suspensions were stained with Fixable viability dye, eBioscience (Cat#65-0865-14 APC-Cy7), and were stained with CD45 (PE/Cy7, Biolegend, CA), CD11b (BV650, Biolegend, CA), F4/80 (BUV395, Biolegend, CA). Following the staining for 30 min in the cold, the cells were washed twice with FACS buffer (PBS + 2% FBS), and the samples were run using fluorescence-activated cell sorter (FACS) Aria II cytometer (BD Biosciences) equipped with a 100 µm nozzle. CD45 + CD11b + F4/80 + live cells were collected in 1.5 mL Eppendorf tubes containing QIAzol Lysis Reagent (Qiagen). Total RNA was isolated using Quick-RNA Microprep Kit (R1050). Bulk RNA sequencing was performed using the Illumina HiSeq 2500. High quality of all samples was confirmed with FastQC v0.11.7.

### Differential gene expression analysis from single-cell RNA sequencing

*PRMT2* expression in myeloid cells from human atherosclerotic plaques between two conditions (diabetic and nondiabetic) was taken from publicly available single-cell RNA sequencing data from Fernandez et al.^22^ and determined using the Seurat function FindMarkers. The Wilcoxon rank-sum test (two-sided) was used to identify *PRMT2* expression between two groups and the logfc.threshold was set to 0.25, and min.cells.group = 25 (minimum of 25% of cells in any group being compared). p-values were adjusted using Bonferroni correction < 0.05**.**

### Data sharing

The RNA seq datasets generated and/or analyzed during the current study are available in the NCBI GEO repository (GSE203523). Additional data generated and/or analyzed during the current study will be available from the corresponding authors on reasonable request.

## Results

### PRMT2 in atherosclerosis regression: study design and metabolic parameters

**Figure 1:**
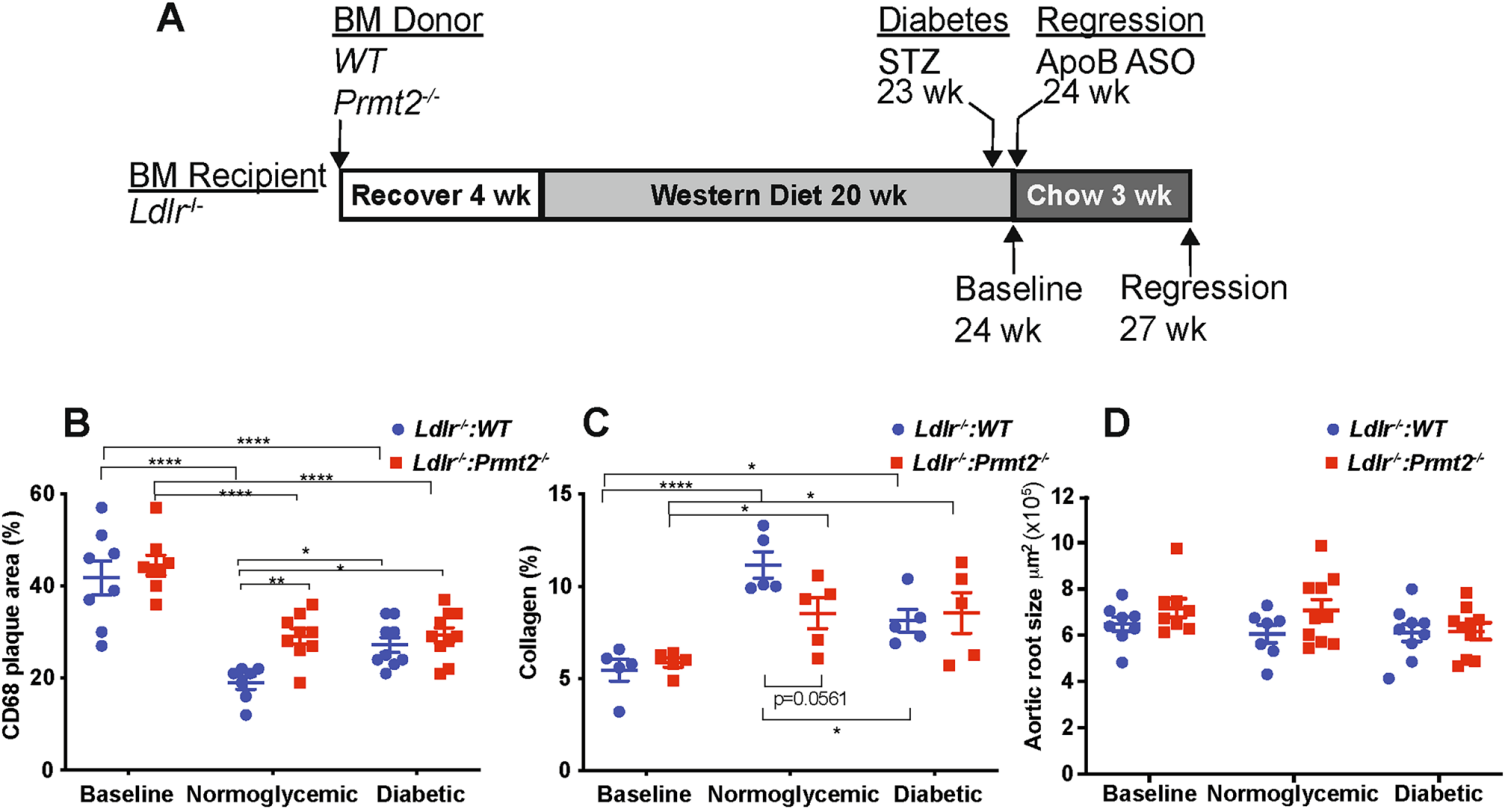
Myeloid PRMT2 deficient mice have increased CD68+ cells and decreased collagen content in nondiabetic conditions after plasma lipid reduction. (**A**) Experimental design: Bone marrow (BM) from WT and *Prmt2*^*−/−*^ donor mice was transplanted into *Ldlr*^*−/−*^ recipient mice. After 4 weeks of recovery, the mice were placed on a Western diet (WD) for 20 weeks. A set of animals at 20 weeks of WD feeding was used as the baseline group. Two other groups of animals received citrate buffer or STZ at 23 weeks. At 24 weeks, to lower lipids and promote regression, mice received apoB-ASO injections, switched to a chow diet for 3 weeks, and were used as the regression groups. (**B**) Aortic roots from baseline and the four regression groups were sectioned and stained for CD68+ cells. Data are shown as the percentage of plaque area. (**C**) Collagen content was determined by picrosirius red staining using both bright field and polarized light microscopy and quantified by Image-Pro Plus software. Data are presented as the percentage of lesion area. (**D**) Total plaque areas were quantified by Image-Pro Plus software. Each group contained 8–10 animals. Data (means ± SEM) were analyzed using one-way ANOVA followed by Bonferroni’s multiple comparison test. *P* values are shown as *P < 0.05 and ****P < 0.001.

To determine the role of PRMT2 in myeloid cells in atherosclerosis regression, we performed bone marrow transplants from littermate WT and *Prmt2*^*−/−*^ mice into lethally irradiated *Ldlr*^*−/−*^ mice, as diagrammed in Fig. 1A. After bone marrow transplant, mice were allowed to recover for 4 weeks and then fed a Western diet (WD) for 20 weeks to permit advanced atherosclerotic plaques to develop. Mice were separated into 6 groups after the dietary period: 2 baseline groups of *Ldlr*^*−/−*^ mice that received either WT or *Prmt2*-deficient bone marrow (designated *Ldlr*^*−/−*^*:WT* and *Ldlr*^*−/−*^:*Prmt2*^*−/−*^, respectively), and four regression groups: (1) normoglycemic WT (NG *Ldlr*^*−/−*^*:WT*); (2) normoglycemic *Prmt2*^*−/−*^ (NG *Ldlr*^*−/−*^:*Prmt2*^*−/−*^); (3) Diabetic WT (D *Ldlr*^*−/−*^*:WT*); and (4) Diabetic *Prmt2*^*−/−*^ (D *Ldlr*^*−/−*^:*Prmt2*^*−/−*^). All regression groups had their apoB-lipoprotein cholesterol levels reduced by injection of an ApoB anti-sense oligonucleotide (ASO) (as done before^23^) and were also switched to a standard chow diet to maximally promote regression. In addition, after 19 weeks of WD feeding, to induce diabetes, *Ldlr*^*−/−*^*:WT* and *Ldlr*^*−/−*^:*Prmt2*^*−/−*^ mice were given daily intraperitoneal injections of STZ for 5 consecutive days (NG mice were injected with citrate buffer)^3,17^.

After 19 weeks of WD, the body weights, total plasma cholesterol, and fasting glucose levels (Supplementary Fig. 1A–C) were similar in the *Ldlr*^*−/−*^ mice reconstituted with WT and *Prmt2*^*−/−*^ bone marrow, as well as the mice randomized into the regression groups (before their treatments). ApoB-ASO treatment resulted in significantly lower plasma cholesterol compared to WT and *Prmt2*^*−/−*^ baseline levels (baseline *Ldlr*^*−/−*^*:WT*: 885 mg/dL, baseline *Ldlr*^*−/−*^*:Prmt2*^*−/−*^*:* 862 to 82–155 mg/dL in the regression groups). Additionally, the reduction in total cholesterol levels was independent of STZ treatment or the source of bone marrow (WT or *Prmt2*^*−/−*^; Supplementary Fig. 1B; see Regression 3 weeks). As expected, the glucose levels were dramatically higher (~ 2.5-fold greater) in STZ-treated mice than in control mice (Supplementary Fig. 1C; Regression 3 weeks D *Ldlr*^*−/−*^*:WT* and D *Ldlr*^*−/−*^*:Prmt2*^*−/−*^).

### Myeloid PRMT2 deficiency phenocopies the impairment in plaque macrophage content reduction in diabetes after lipid lowering

To determine the effect of myeloid PRMT2 status on atherosclerosis regression, plaque area and the contents (as a % of plaque area) of macrophages (CD68 + cells) and collagen (Sirius red + area under polarized light) were measured. Both groups of baseline mice had similar macrophage plaque areas and collagen contents (Fig. 1B,C; Supplementary Fig. 2). After hyperlipidemia was reduced, although plaque areas remained similar to baseline values (Fig. 1D), there were significant changes in plaque composition. This implies that as in other contexts, the effects of a given factor, in this case PRMT2 status, operate within a certain range of plasma cholesterol^24^, with elevated plasma levels being dominant over myeloid PRMT2 deficiency. Reversal of hyperlipidemia, however, apparently allowed PRMT2 deficiency to become penetrant, based on the changes in plaque composition. There was a significant reduction in plaque macrophage content in the NG *Ldlr*^*−/−*^*:WT* group. Although there was also a reduction in the NG *Ldlr*^*−/−*^:*Prmt2*^*−/−*^ group, it was attenuated to a similar degree as we have observed in diabetic mice^17,24,25^ and as in the present study (compare the data in Fig. 1B between NG *Ldlr*^*−/−*^:*Prmt2*^*−/−*^ and D *Ldlr*^*−/−*^*:WT*). In *Ldlr*^*−/−*^:*Prmt2*^*−/−*^ mice, however, plaque macrophage content was similar in both the normoglycemic and diabetic groups (Fig. 1B; Supplementary Fig. 2), suggesting that with myeloid deficiency of PRMT2, regression became independent of diabetes.

In contrast to the compositional changes, plaque areas (aortic root size) among the groups were not statistically significant (Fig. 1C). Consistent with our previous findings, the decreases in plaque macrophage content were mirrored by corresponding increases in collagen content (Fig. 1B; Supplementary Fig. 2), presumably because of less degradation of collagen by macrophages with a lower level of activation (see below for data to support this presumption). The increases in collagen content were lower in the NG *Ldlr*^*−/−*^:*Prmt2*^*−/−*^, D *Ldlr*^*−/−*^:*Prmt2*^*−/−*^, and D *Ldlr*^*−/−*^:*WT* groups, as expected from their more modest changes (vs. NG *Ldlr*^*−/−*^:*WT* mice) in plaque macrophage content.

As noted in the Introduction, hyperglycemia was associated with decreased expression of *Prmt2* in macrophages in vitro. To confirm that this was also true in vivo, in a separate set of mice, we measured *Prmt2* mRNA levels in plaque macrophages in normoglycemic and hyperglycemic mice. The levels of *Prmt2* mRNA in plaque macrophages (F4/80^+^ cells selected from aortic digestion by FACS) from diabetic mice were ~ 50% of those in normoglycemic mice (Supplementary Fig. 3A). Consistent with this finding are the data from myeloid cells in human plaques (originally reported in Ref.^22^), in which the expression of *PRMT2* mRNA was statistically lower in samples taken from those with diabetes (Supplementary Fig. 3B). Together with the data in Fig. 1, the results suggest that loss of PRMT2 expression either by genetic deficiency or by repression by hyperglycemia impairs the regression of plaque macrophage content after lipid lowering.

### PRMT2 deficiency increases macrophage retention, but does not change monocyte recruitment to, or macrophage proliferation in, plaques

**Figure 2:**
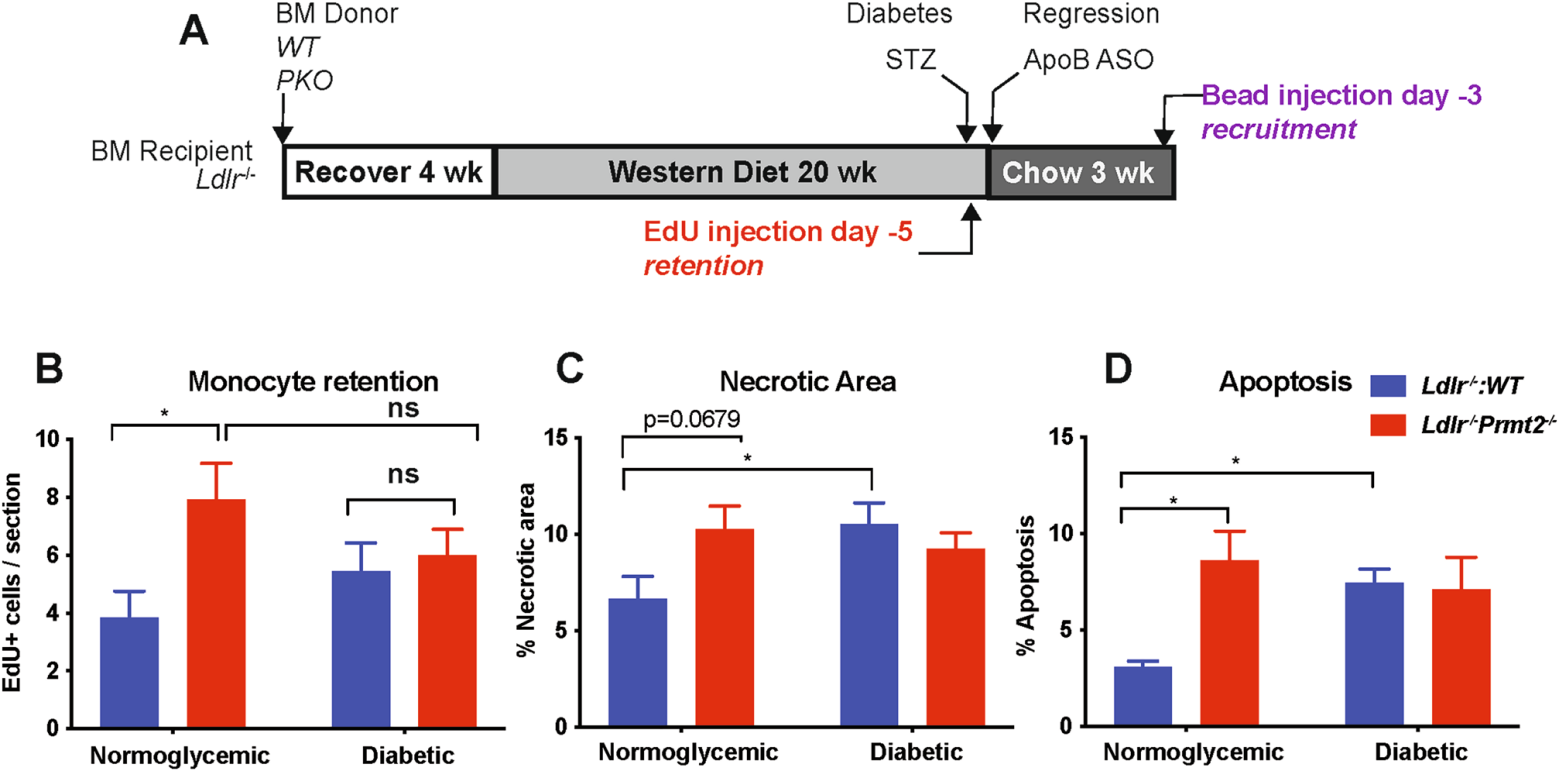
Macrophage dynamics show that monocyte retention is the key kinetic change impairing regression in *Ldlr*^*−/−*^*:Prmt2*^*−/−*^ mice in normoglycemia. (**A**) Schematic of timeline for EdU and bead injections to assess retention and recruitment of monocytes, respectively, into aortic roots under regression conditions. (**B**) Analysis of EdU: CD68+ cells/section of atherosclerotic plaques from the indicated groups showing significantly increased recruitment of EdU+ cells into *Ldlr*^*−/−*^*:Prmt2*^*−/−*^ compared to *Ldlr*^*−/−*^*:WT* under normoglycemic conditions. Plaques were also stained for CD68 and the number of EdU+/CD68+ cells was measured. (**C**) Quantification of necrotic area and (**D**) cleaved caspase 3 (to assess apoptosis) in plaque sections showing increased necrosis and apoptosis in *Ldlr*^*−/−*^*:Prmt2*^*−/−*^ vs *Ldlr*^*−/−*^*:WT* under normoglycemic conditions. Data (means ± SEM) were analyzed using one-way ANOVA followed by Bonferroni’s multiple comparison test. *P < 0.05.

To investigate the basis for the difference in macrophage content between *Ldlr*^*−/−*^:*WT* and *Ldlr*^*−/−*^:*Prmt2*^*−/−*^ regression groups, we examined key kinetic parameters that regulate plaque monocyte/macrophage abundance including recruitment, retention, proliferation, apoptosis and necrosis as we have done before^17,26–29^. The experimental design is shown in Fig. 2A. To assess monocyte recruitment during the regression period, mice were injected with fluorescent latex beads 3 days before harvest, and the number of beads in the aortic plaque was quantified and normalized to the efficiency of labeling circulating monocytes. The efficiency of monocyte labeling was not affected by either hyperglycemia or PRMT2 deficiency (not shown).

Monocyte recruitment was not significantly different between the regression groups (NG *Ldlr*^*−/−*^:*WT*, NG *Ldlr*^*−/−*^:*Prmt2*^*−/−*^, D *Ldlr*^*−/−*^:*WT*, D *Ldlr*^*−/−*^:*Prmt2*^*−/−*^) (Supplementary Fig. 4A). Diabetes did not significantly increase monocyte recruitment in WT mice (Supplementary Fig. 4A). Thus, we turned to retention/emigration as a possible mechanism as the basis for the greater plaque macrophage content in the other 3 regression groups compared to the NG WT group.

To determine whether macrophage retention was relatively increased (reflecting a decreased capacity to emigrate) in the non-NG WT groups, mice were injected with EdU (5-ethynyl-2′-deoxyuridine) 5 days before the induction of regression to mark cells that accumulate during plaque formation. We have previously reported^28^ that EdU preferentially labels circulating Ly6c^hi^ monocytes^28^, which are thought to be the major contributors to the plaque macrophage pool. We then compared the abundance of EdU+; CD68+ cells between baseline and the regression groups to characterize the subset of monocytes that are leaving the plaque. The number of EdU-positive cells was normalized to the EdU labeling efficiency in blood monocytes by flow cytometry and was similar between the *Ldlr*^*−/−*^:*WT and Ldlr*^*−/−*^:*Prmt2*^*−/−*^ baseline groups. Compared to the baseline groups (Supplementary Fig. 4), which on average showed 32 EdU+; CD68+ cells per section, there was a decrease in EdU+; CD68+ cells in all regression groups, suggesting that cells were actively leaving the plaque during regression. We found, however, that EdU+ macrophages were significantly more abundant in NG *Ldlr*^*−/−*^:*Prmt2*^*−/−*^ mice than in NG *Ldlr*^*−/−*^:WT mice (Fig. 2B). Furthermore, the EdU+ macrophage abundance in the NG *Ldlr*^*−/−*^:*Prmt2*^*−/−*^ mice was not significantly different from that in either diabetes group (Fig. 2B). Thus, PRMT2-deficiency reduced the ability of macrophages to migrate out of the plaque under normoglycemic conditions, potentially contributing to the impaired regression of atherosclerosis.

To understand whether macrophage proliferation affected plaque macrophage content, Ki67 (a marker of cell proliferation) staining was performed (Supplementary Fig. 4B). We observed that the number of Ki67 cells did not change among the regression groups, further supporting changes in macrophage retention/emigration as the basis for the increased CD68+ cell content in the NG *Ldlr*^*−/−*^:*Prmt2*^*−/−*^, D *Ldlr*^*−/−*^:*Prmt2*^*−/−*^, and D *Ldlr*^*−/−*^:*WT* regression groups. This is in contrast to data in vascular injury models that PRMT2 deficiency promoted vascular smooth muscle cell proliferation^30,31^, which likely reflects differential effects of PRMT2 by cell type or by perturbation. Furthermore, once myeloid cells are deficient in PRMT2, an additional effect of diabetes was not observed, implying that a major effect of hyperglycemia is mediated through its downregulation of *Prmt2*.

### Effects of myeloid *Prmt2* deficiency and diabetes on necrotic area and apoptosis

In addition to macrophage content, another measure of disease severity in atherosclerosis is the size of the necrotic core, especially because of its association in humans with plaque rupture^32,33^. Thus, we quantified the acellular areas (taken to reflect necrotic areas^24^) in aortic plaques. As shown in Fig. 2C, the necrotic area was lowest in the NG *Ldlr*^*−/−*^:*WT* group. Notably, the higher necrotic area in the NG *Ldlr*^*−/−*^:*Prmt2*^*−/−*^ mice was similar to that in D *Ldlr*^*−/−*^:*Prmt2*^*−/−*^ mice (as well as in the D *Ldlr*^*−/−*^:*WT* mice). As with the plaque macrophage content data (Fig. 1B) and retention data (Fig. 2B), diabetes did not exacerbate the effects of PRMT2-deficiency, again implying that another major effect of hyperglycemia is mediated through its downregulation of PRMT2.

The acellular area is thought to result from the apoptosis of macrophages. When we used caspase-3 staining to determine apoptosis, we found that the pattern paralleled the necrotic core area results (Fig. 2D), namely that the level of apoptosis was lowest in the NG *Ldlr*^*−/−*^:*WT* group, with similar (elevated) levels in the remaining 3 regression groups. Thus, the loss of PRMT2 expression by genetic deletion under normoglycemic conditions or upon reduced expression in diabetes promotes apoptosis resulting in a larger necrotic area. This effect in myeloid cells is in contrast to PRMT2 deficiency decreasing apoptosis in fibroblasts^12^, and, again, may reflect differential effects of cell type or perturbations.

### Loss of PRMT2 promotes the expression of genes linked to cytokine signaling and inflammatory pathways in plaque CD68+ cells under normoglycemic conditions

**Figure 3:**
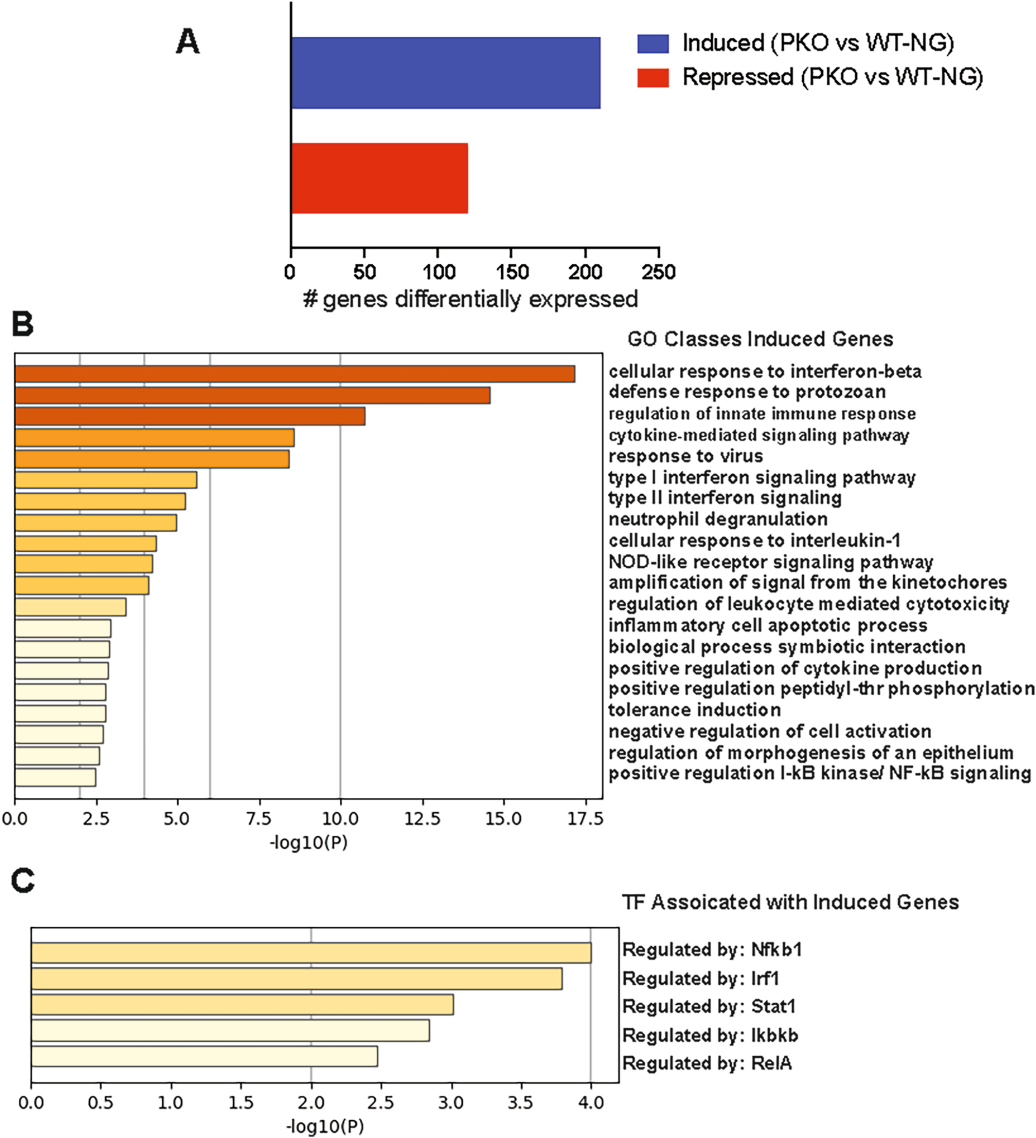
GO classes and transcription factors associated with genes upregulated in *Ldlr*^*−/−*^*:Prmt2*^*−/−*^ plaque CD68+ cells under normoglycemic conditions. (**A**) PRMT2 deficiency in plaque CD68+ cells resulted in the upregulation of 204 and downregulation of 120 genes (logFC < 2.0, *P* < 0.01). (**B**) Metascape analysis of pathways for genes up-regulated in *Ldlr*^*−/−*^*:Prmt2*^*−/−*^ reveals genes involved in cytokine signaling and innate immune/inflammatory responses. (**C**) Transcription factors associated with the genes upregulated in *Ldlr*^*−/−*^*:Prmt2*^*−/−*^ in normoglycemia include the prototypical proinflammatory transcriptional regulatory factors NF-kappa B, IRF1, and STAT1.

To gain insight into why PRMT2-deficient myeloid cells fail to regress atherosclerosis compared to WT cells under nondiabetic conditions, we examined gene expression changes by performing bulk RNA sequencing (RNA-seq) on NG *Ldlr*^*−/−*^:WT and NG *Ldlr*^*−/−*^:*Prmt2*^*−/−*^ plaque CD68+ cells collected by laser-capture microdissection^20^. We found that in nondiabetic conditions, CD68 + *Ldlr*^*−/−*^:*Prmt2*^*−/−*^ cells had induced 204 and repressed 120 genes compared to *Ldlr*^*−/−*^:WT cells (logFC < 2.0, *P* < 0.01) (Fig. 3A). Metascape^21^ pathway analysis indicated that the upregulated genes in *Ldlr*^*−/−*^:*Prmt2*^*−/−*^ CD68+ cells were involved in cytokine signaling and inflammatory/innate immune responses (Fig. 3B). Moreover, analysis of the interactions between transcription factors (TFs) and the gene targets using the TRRUST (transcriptional regulatory relationships revealed by sentence-based text-mining) algorithm^34^ identified NF-kappa B, followed by IRF1 and STAT1, as the top TFs controlling gene expression in *Ldlr*^*−/−*^:*Prmt2*^*−/−*^ vs *Ldlr*^*−/−*^:*WT* CD68+ cells under normoglycemic conditions (Fig. 3C).

The plaque expression profile under regressing and nondiabetic conditions revealed genes encoding proteins that mediate cellular adhesion, (*Mpz*, *Tnr*, *Frem1*, and *Iigp1*) and inflammation (*Alox12, Tnf*, *Cxcl5,* and *Cxcl2)* were up-regulated in *Prmt2*^*−/−*^ CD68+ cells compared to WT (Table 1). We previously identified *Cxcl5 and Cxcl2* as upregulated in regressing plaques^27^. This suggests that upon lipid lowering in the absence of PRMT2, the CD68+ cells in the regressing plaque take on more adherent and inflammatory characteristics.

**Table 1:** Genes upregulated in *Prmt2*^*−/−*^ compared to WT regressing plaque CD68+ cells associated with cell adhesion and inflammation.

Gene	Log^2^ fold (*Prmt2*^*−/−*^/WT)	p-value	Function
*Mpz*	7.5	4.1 E−07	Cell adhesion molecule
*Tnr*	3.9	3.1 E−04	Extracellular matrix protein
*Frem1*	3.2	7.9 E−04	Extracellular matrix protein
*Iigp1*	3.1	3.5 E−04	Cell adhesion molecule
*Alox12*	5.6	1.3 E−05	Produces proinflammatory Lipid mediators
*Cxcl5*	5.6	1.5 E−05	Proinflammatory cytokine
*Cxcl2*	3.8	3.2 E−04	Proinflammatory cytokine
*Tnf*	2.3	2.7 E−03	Proinflammatory cytokine

Expression change from RNA-seq data.

The top GO class associated with the genes repressed in *Ldlr*^*−/−*^:*Prmt2*^*−/−*^ compared to *Ldlr*^*−/−*^:*WT* CD68+ cells under normoglycemia is the regulation of pseudopodium assembly (Supplementary Fig. 5A). Pseudopodia are used by macrophages for phagocytosis^35^. The top transcription factor associated with the genes reduced upon *Prmt2* deletion was EGR1 (Supplementary Fig. 5B). EGR1 has been shown to occupy enhancers associated with inflammatory response genes to decrease their expression^36^.

The RNA sequencing results showed that PRMT2 deficient plaque macrophages had a more inflammatory transcriptional profile, which is consistent with its reported role in regulating the inflammatory responses^12,13^. Specifically, in fibroblasts, PRMT2 has been shown to be an inhibitor of NF-kappa B and its deficiency dampens lung macrophage responsiveness to LPS^12,13^. To investigate whether PRMT2 plays similar roles in our system, BMDMs were isolated from either *Prmt2*-sufficient or *Prmt2*-deficient mice and maintained during their 7-day differentiation period under metabolic conditions to simulate normoglycemia (5 mM d-glucose) or hyperglycemia (25 mM d-glucose). As a control for the osmolality difference, the medium that was 5 mM d-glucose was supplemented with 20 mM l-glucose. Cells were then treated with either IL-4 to promote M2 polarization, or LPS to induce the M1 state. We then measured the expression of standard markers of macrophages responding to inflammation resolving IL-4 (*Arg1* and *Fizz1)* and inflammation-promoting LPS (*Il6* and *Nos2*) as a function of normal glucose and high glucose, and in either the absence or presence of PRMT2.

**Figure 4:**
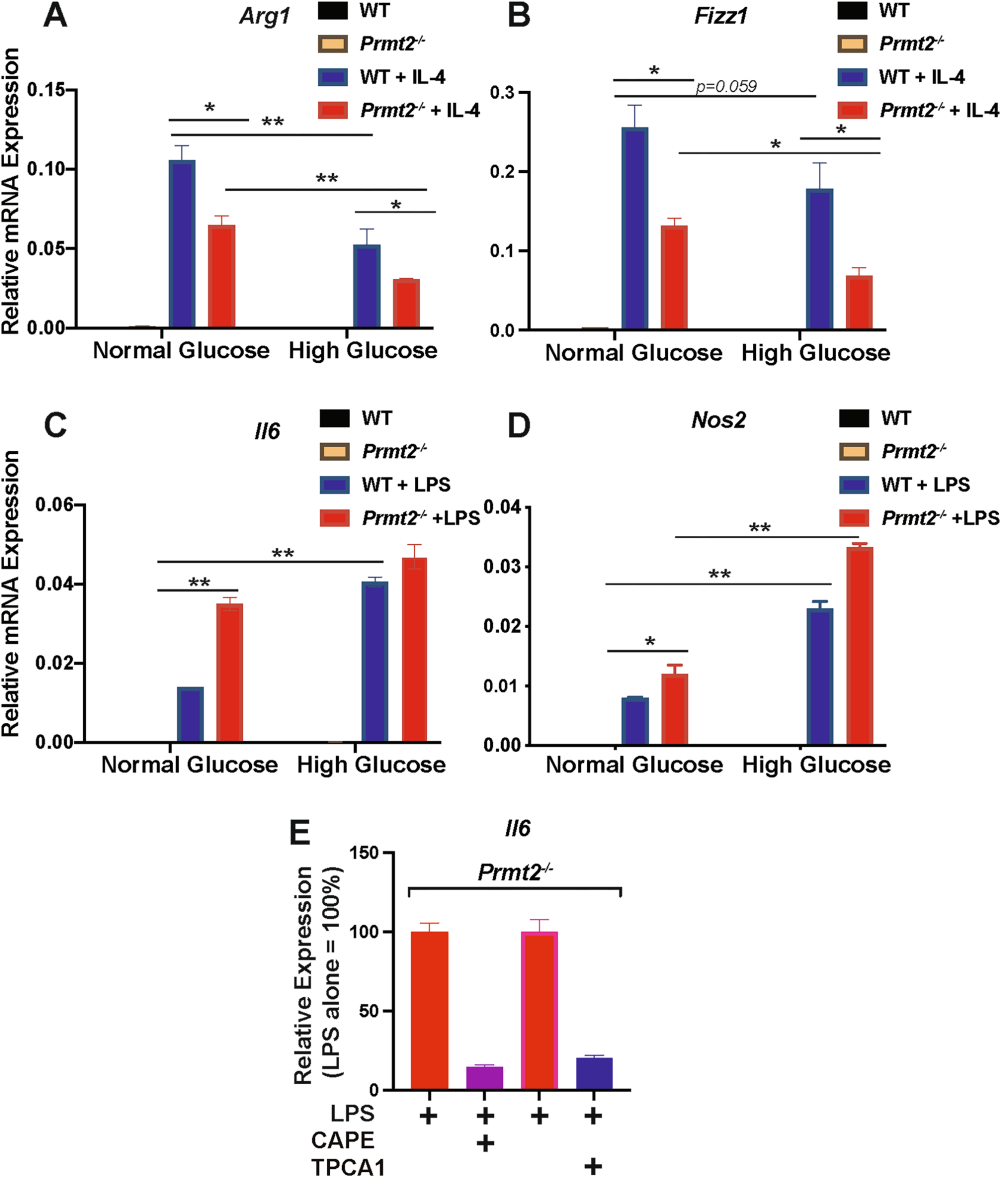
PRMT2 deficiency blunted the macrophage response induced by IL-4 and heightened the macrophage response to LPS in vitro. BMDMs from WT and *Prmt2*^*−/−*^ mice were differentiated into M0 (unactivated) macrophages in normal d + l-glucose (100 mg/dL d-glucose + 350 mg/dL l-glucose) or high d-glucose (450 mg/dL) for 7 days, and then treated with or without IL-4 (to induce M2 polarization) or with or without LPS (to induce M1 polarization). RNA was prepared and qPCR was performed on the M2 marker genes (**A**) arginase 1 (*Arg1*) and (**B**) *Fizz1*, and the M1 markers (**C**) *Il6* and (**D**) *Nos2*, and expression normalized to cyclophilin A is shown (n = 3). Data (means ± SEM) were analyzed using one-way ANOVA followed by Bonferroni’s multiple comparison test. *p < 0.05 and **p < 0.001. (**E**) LPS induction of *Il6* is NF-kappa B dependent. *Prmt2*^*−/−*^ BMDMs were differentiated in normal glucose as above. Cells were pretreated for 2 h with NF-kappa B inhibitors CAPE (5 μM), or TPCA1 (5 μM) and then treated with LPS for 6 h. RNA was isolated and *Il6* expression was determined by qPCR relative to cyclophilin A1 and is shown as percent relative to untreated cells, which was set to 100%. The data presented are means with error bars representing the range of the means from an experiment that was repeated twice.

We had previously reported that responsiveness to IL-4, which promotes inflammation resolution, is required for plaque regression^28^ and that hyperglycemia attenuates macrophage responsiveness to IL-4^17^. Whereas the induction of *Arg1* and *Fizz1* by IL-4 was blunted, as expected, by hyperglycemia, the effect of PRMT2-deficiency in BMDMs is similar to that of hyperglycemia in that the levels of *Arg1* and *Fizz1* were lower than the corresponding values for the sufficient cells (Fig. 4A,B). Thus, the loss of PRMT2 in normal glucose mimics the high glucose state.

As noted above, PRMT2 is a known inhibitor of NF-kappa B with a mechanism involving blocking nuclear export of I kappa B-alpha, resulting in decreased NF-kappa B binding to its transcriptional targets^12^. Thus, we hypothesize that the increases in the expression of targets of NF-kappa B would result from the relief of this inhibition. To test this hypothesis, we stimulated WT and *Prmt2*^*−/−*^ BMDMs with LPS and measured the expression of the NF-kappa B target genes *Il6* and *Nos2*. We found that high glucose compared to normal glucose increased *Il6* and *Nos2* expression, and this was further amplified in *Prmt2*^*−/−*^ BMDMs compared to WT BMDMs (Fig. 4C,D). To demonstrate a direct role of NF-kappa B in the heightened inflammatory state of PRMT2-deficient BMDMs, we also examined whether the induction of *Il6* by LPS was reduced upon inhibition of NF-kappa B by caffeic acid phenethyl ester (CAPE), an inhibitor that blocks NF-kappa B binding to DNA^37^, or TPCA1^38,39^, a potent and selective inhibitor of IκB kinase 2 (IKK-2) required for NF-kappa B activation^40^. When either NF-kappa B inhibitor was used the increase in *Il6* under normal glucose conditions in *Prmt2*^*−/−*^ BMDMs was attenuated (Fig. 4E). Taken together, these data are consistent with the RNA-seq analysis that suggested that PRMT2 deficiency heightens the inflammatory state of plaque macrophages, and suggests that the acquisition of an inflammatory phenotype by the loss of PRMT2 occurs through activation of NF-kappa B. Furthermore, PRMT2-deficiency not only increases responsiveness to an inflammatory stimulus, but also blunts the response of macrophages to the potent inflammation resolving cytokine, IL-4.

## Discussion

Diabetes clinically impairs the benefits of cholesterol lowering^1^, and we have previously shown results consistent with this in a mouse model of atherosclerosis regression^17,24,25,41^. When probing the molecular basis for these adverse effects of diabetes, we found that elevated glucose represses the expression of PRMT2^6^, leading to the present studies to test the hypothesis that some of the effects of diabetes are a consequence of this repression. Indeed, the data presented support this hypothesis, particularly by the impairment in the desirable remodeling of the plaque composition after cholesterol lowering in mice with myeloid deficiency of PRMT2 that were normoglycemic. The ability of PRMT2 deficiency to phenocopy the effects of diabetes in the regression, but not progression, setting implies that it would be more of an important factor in people undergoing risk factor reduction than during the unabated progression of their disease.

It has been reported that hyperglycemia promotes monocytosis^3^. However, in our bone marrow transplant model, we did not observe any significant increase in the levels of circulating monocytes under any of our experimental conditions, so monocytosis is unlikely to contribute to the phenotype. The most plausible molecular mechanism for the impaired resolution of inflammation required for maximal responses to cholesterol lowering in mice^28^ and people (CANTOS)^42^, is the inhibition of NF-kappa B by PRMT2.

We initially identified PRMT2 as a factor that enhanced LXR ligand-dependent expression of *Abca1* in normal glucose^6^. However, we did not observe changes in *Abca1* expression by RNA-seq on CD68+ cells from the regressing plaques under nondiabetic conditions. This likely reflects the lack of LXR ligands (oxysterols) present in the plaque environment upon cholesterol lowering during the 3-week regression period after which CD68+ cells were selected, thereby resulting in little activation of *Abca1.*

Although PRMT2 appears to play a major role in the regression of atherosclerosis in diabetes, other factors have been reported to either enhance or repress the regression of atherosclerosis in diabetes. For example, ectopic expression of human aldose reductase in mice has been shown to amplify the impaired regression of atherosclerosis in diabetic mice by directing glucose into pathways producing inflammatory metabolites^43^. In addition, when the receptor for the advanced glycation end-products (RAGE) is deleted in diabetic mice, it accelerates the regression of atherosclerosis^44^. Thus, factors that sense and respond to hyperglycemia affect the regression of atherosclerosis in mouse models of diabetes. Whether PRMT2 functionally interacts with these pathways by catalyzing asymmetric arginine dimethylation of pathway components to affect their function is not known. This highlights the importance of identifying PRMT2 substrates in macrophages.

Our study has limitations: namely, the mouse model of diabetes we have employed is Type 1, whereas the majority of diabetes in humans is Type 2^45^. It should be noted, however, that CHD is the leading cause of death in people with either form of diabetes^46,47^. Nonetheless, it would be interesting in future studies to determine whether the regression of atherosclerosis is also impaired in dietary or genetic mouse models of Type 2 diabetes and whether this is associated with reduced PRMT2 expression in plaque macrophages.

With the availability of curated data sets from human atherosclerotic plaques from people with and without diabetes^22^ we were able to extend our mouse model findings to a relevant clinical scenario. Indeed, we observed lower *PRMT2* expression in myeloid cells of plaques from humans with diabetes compared to those without diabetes, supporting the relevance of our findings in mouse models to human atherosclerosis. Our study is the first to illustrate a direct link between PRMT2 and the regression of atherosclerosis in diabetes and represents exciting progress in the study of arginine methyltransferases in CHD.

## Supplementary Information

Supplementary Information 1.

Supplementary Information 2.

## Abbreviations

PRMT2: Protein arginine methyltransferase 2
Ldlr: Low density lipoprotein receptor
CHD: Coronary heart disease
LXR: Liver X receptor
ASO: Anti-sense oligonucleotide
ApoB: Apolipoprotein B
