# Supplementary material for: Loss of PRMT2 in myeloid cells in normoglycemic mice phenocopies impaired regression of atherosclerosis in diabetic mice
Source: Sci Rep. 2022 Jul 14;12:12031. doi: 10.1038/s41598-022-15349-6 (PMC9283439; doi:10.1038/s41598-022-15349-6)
Supplement: Supplementary file 1 — Supplementary Information 1. [file 41598_2022_15349_MOESM1_ESM.docx]

**Supplementary Figure 1. Plasma cholesterol concentrations, blood glucose levels, and weights from *Ldlr^-/-^:WT* and *Ldlr^-/-^:Prmt2^-/-^* cohorts**

A) Plasma total cholesterol concentrations, B) glucose levels, and C) body weight were measured at the 19-week time point of Western diet feeding for the baseline groups and at the end of the experiment for the indicated regression groups under conditions of normoglycemia and diabetes.

**Supplementary Figure 2. Collagen content in plaques from *Ldlr^-/-^:WT* and *Ldlr^-/-^:Prmt2^-/-^ mice* with and without diabetes**

A) Representative micrographs of collagen content as determined by picrosirius red staining and imaged using brightfield and polarized light microscopy. B) Quantification of picrosirius red staining.

**Supplementary Figure 3. Expression of PRMT2 in plaque macrophages from mice and myeloid cells from human atherosclerotic plaques.**

A) The levels of *Prmt2* mRNA in plaque macrophages cells selected from aortic digestion by FACS(CD45+/Cd11b+/F4/80^+^) from diabetic compared to normoglycemic mice. B) Single-cell RNA sequencing of myeloid cells from human atherosclerotic plaques from nondiabetic and diabetic (Type 2) patients were analyzed for the expression of *PRMT2* (n=6). Data were acquired from publicly available single cell sequencing data from Fernandez *et al* ^22^.

**Supplementary Figure 4. Monocyte recruitment and macrophage proliferation in plaques are not significantly different between *Ldlr^-/-^:WT* and *Ldlr^-/-^:Prmt2^-/-^* mice under normoglycemic and diabetic conditions**

A) Analysis of bead^+^ cells/section of atherosclerotic plaques is shown for each group. B) Quantification of Ki67 staining in plaque sections to assess proliferation. No significant differences were observed in either monocyte recruitment or proliferation between the recipient groups.  C) Numbers of EdU+; CD68+ cells/section from *Ldlr^-/-^:WT* and *Ldlr^-/-^:Prmt2^-/-^* at baseline (before regression) shows no significant change in EdU labeling between genotypes.

**Supplementary Figure 5. GO classes and transcription factors associated with genes downregulated in PRMT2-deficient plaque CD68+ cells under normoglycemic conditions**

A) Metascape analysis of the 120 genes downregulated in *Ldlr^-/-^:Prmt2^-/-^* plaque CD68+ cells compared to *Ldlr^-/-^:WT* cells reveals pathways involved in pseudopodium assembly, among others. B) Transcription factor EGR1 is associated with the genes downregulated in *Ldlr^-/-^:Prmt2^-/-^* normoglycemia.
