# Supplementary material for: Loss of PRMT2 in myeloid cells in normoglycemic mice phenocopies impaired regression of atherosclerosis in diabetic mice
Source: Sci Rep. 2022 Jul 14;12:12031. doi: 10.1038/s41598-022-15349-6 (PMC9283439; doi:10.1038/s41598-022-15349-6)

Supplementary Figure 1

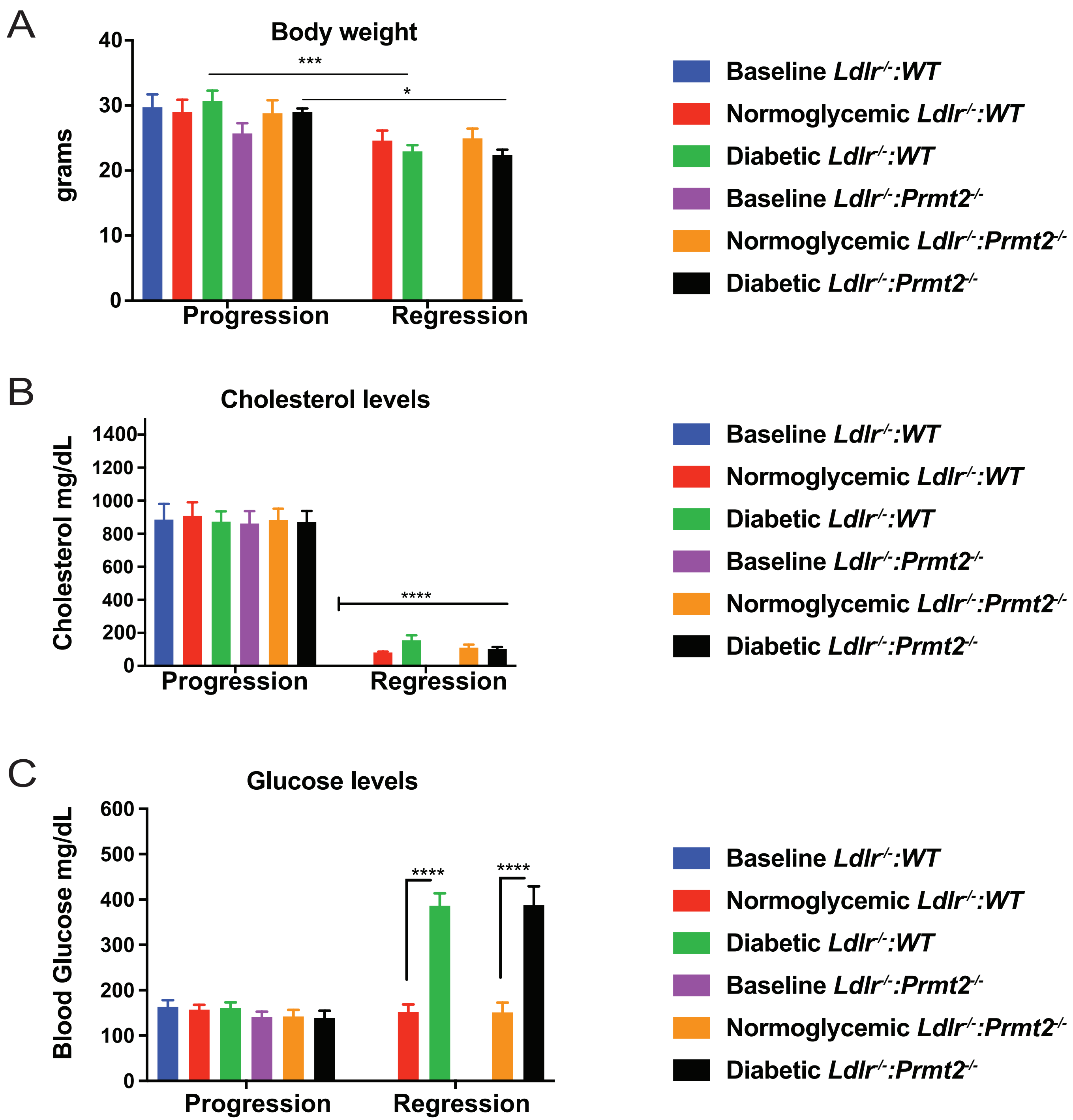

Supplementary Figure 2

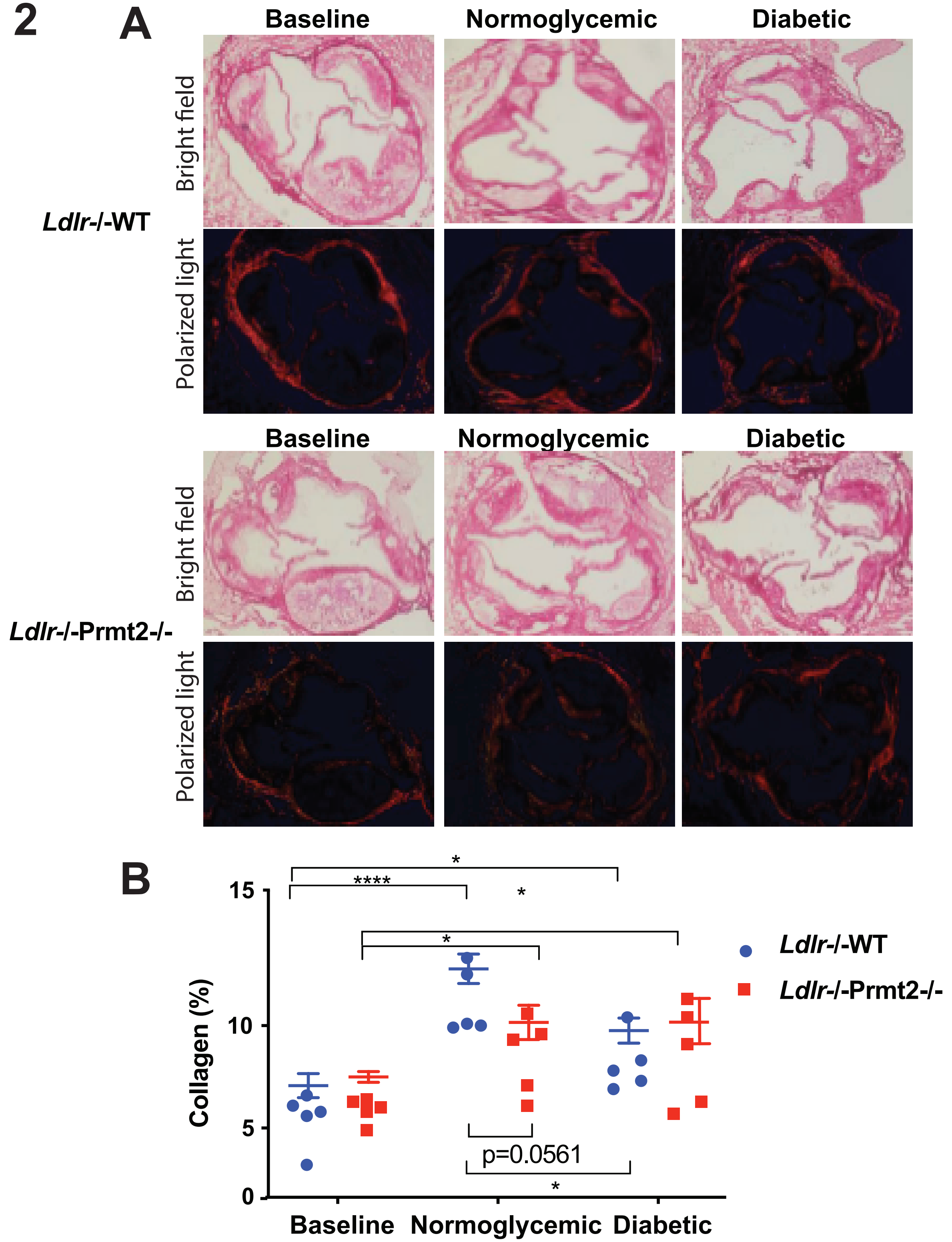

Supplementary Figure 3

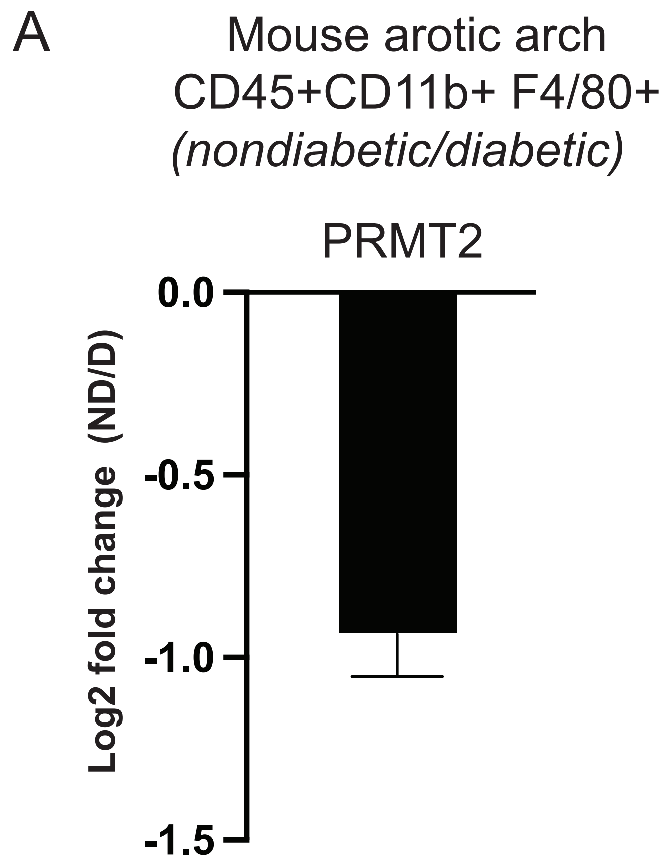

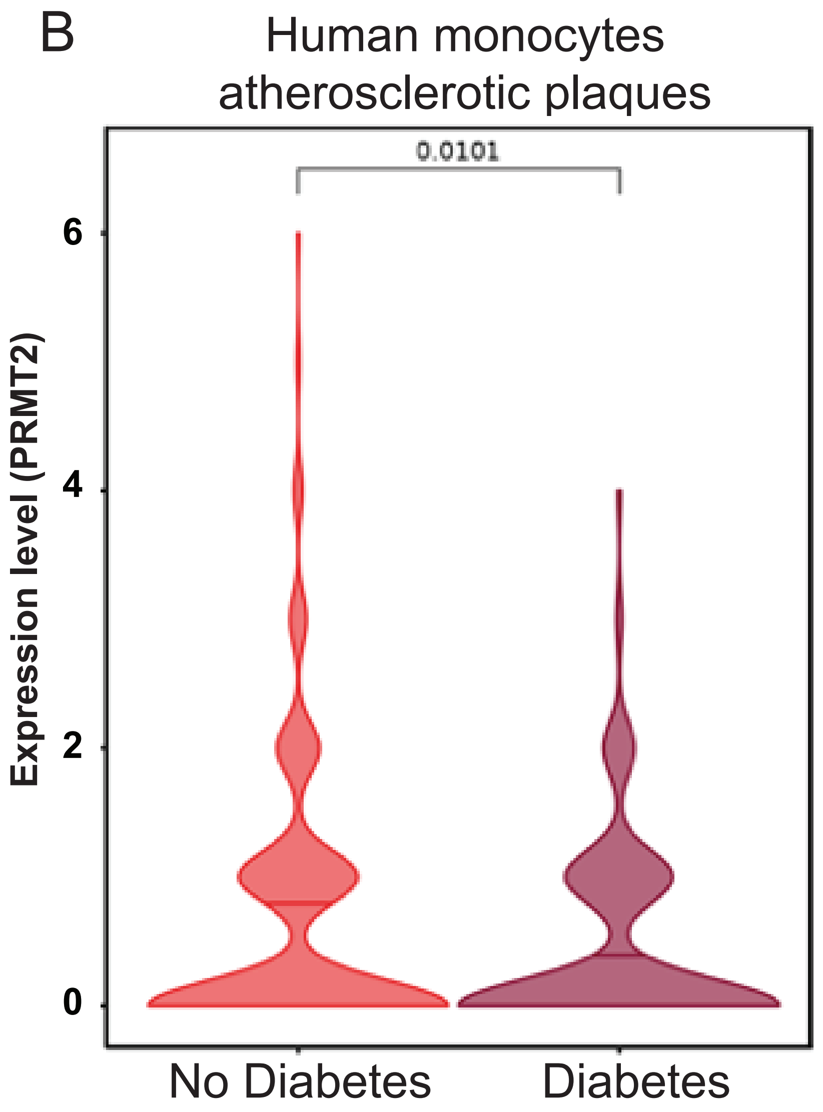

Supplementary Figure 4

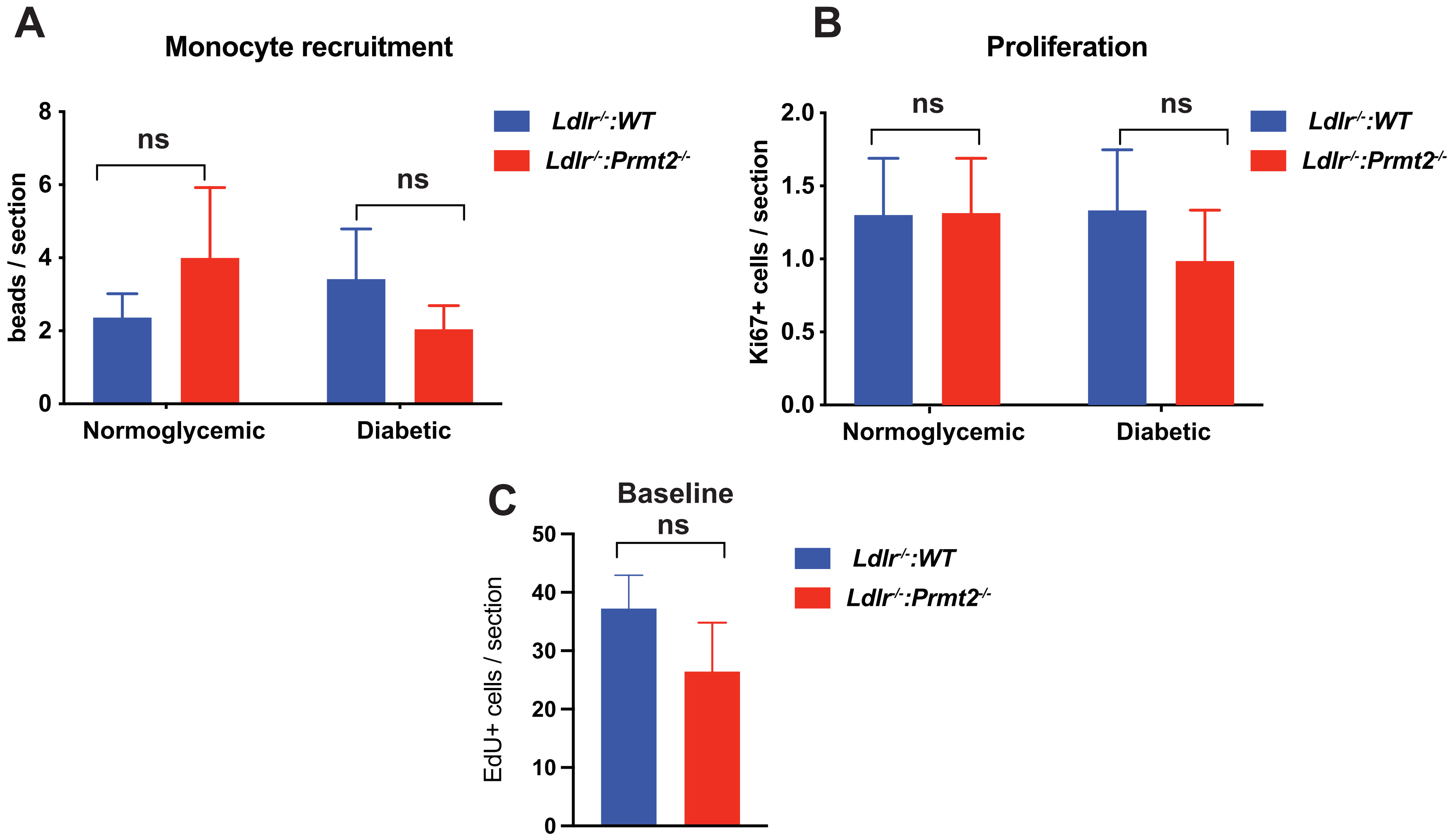

Supplementary Figure 5

A

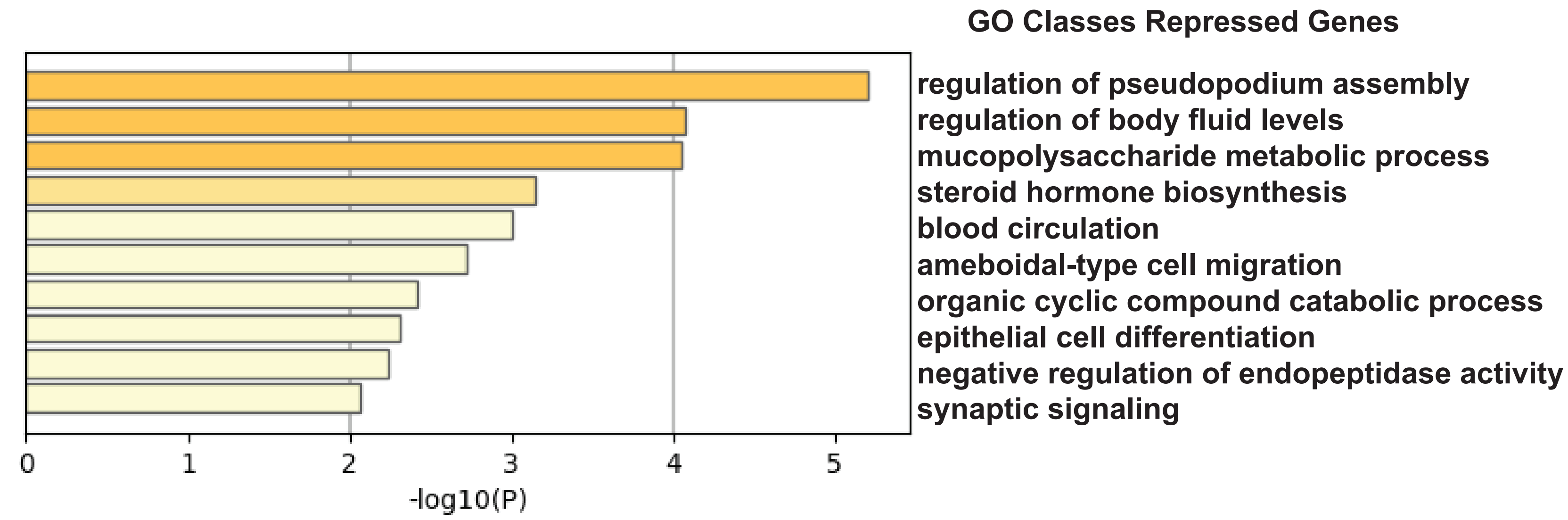

B

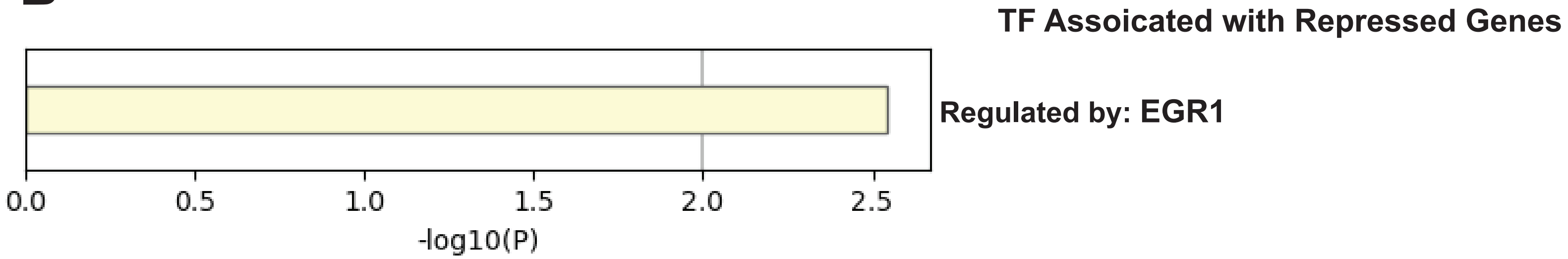

Supplement: Supplementary file 2 — Supplementary Information 2. [file 41598_2022_15349_MOESM2_ESM.pdf]
